# Supplementary material for: Changes in Lifestyle Behaviors, Shopping Habits and Body Weight Among Adults in Cyprus and Greece During COVID-19 Lockdown: A Cross-Sectional Study
Source: Nutrients. 2025 Jan 8;17(2):214. doi: 10.3390/nu17020214 (PMC11767316; doi:10.3390/nu17020214)
Supplement: Supplementary file 1 [file nutrients-17-00214-s001.zip › nutrients-3418951-supplementary.pdf]

STROBE Statement—checklist of items that should be included in reports of ***cross sectional studies***

	Item No	Recommendation	Paragraph/ Section
**Title and abstract**	1	(*a*) Indicate the study's design with a commonly used term in the title or the abstract	Study design;
		(*b*) Provide in the abstract an informative and balanced summary of what was done and what was found	Objective; Conclusions
**Introduction**			
Background/rationale	2	Explain the scientific background and rationale for the investigation being reported	Paragraphs 1-2
Objectives	3	State specific objectives, including any prespecified hypotheses	Paragraph 6
**Methods**			
Study design	4	Present key elements of study design early in the paper	Paragraph 1
Setting	5	Describe the setting, locations, and relevant dates, including periods of recruitment, exposure, follow-up, and data collection	Paragraph 2-8
Participants	6	(*a*)*Cross-sectional study*—Give the eligibility criteria, and the sources and methods of selection of participants	Paragraph 2-3
Variables	7	Clearly define all outcomes, exposures, predictors, potential confounders, and effect modifiers. Give diagnostic criteria, if applicable	Section 2.4
Data sources/ measurement	8*	For each variable of interest, give sources of data and details of methods of assessment (measurement). Describe comparability of assessment methods if there is more than one group	*Section 2.2,2.3*
Bias	9	Describe any efforts to address potential sources of bias	Paragraph 3
Study size	10	Explain how the study size was arrived at	Paragraph 2-3
Quantitative	11	Explain how quantitative	Section2.4

variables		variables were handled in the analyses. If applicable, describe which groupings were chosen and why	
Statistical methods	12	(*a*) Describe all statistical methods, including those used to control for confounding	Section 2.4
		(*b*) Describe any methods used to examine subgroups and interactions	Section 2.4
		(*c*) Explain how missing data were addressed	Section 2.4
		(*d*) *Cross-sectional study*—If applicable, describe analytical methods taking account of sampling strategy	NA
		(*e*) Describe any sensitivity analyses	NA

Continued on next page

**Results**			
Participants	13*	(a) Report numbers of individuals at each stage of study—eg numbers potentially eligible, examined for eligibility, confirmed eligible, included in the study, completing follow-up, and analysed	Methods Par.1
		(b) Give reasons for non-participation at each stage	Paragraph 1
		(c) Consider use of a flow diagram	None
Descriptive data	14*	(a) Give characteristics of study participants (eg demographic, clinical, social) and information on exposures and potential confounders	Table 1,1a,2
		(b) Indicate number of participants with missing data for each variable of interest	NA
Outcome data	15*		
		Cross-sectional study—Report numbers of outcome events or summary measures	Table 1-9
Main results	16	(*a*) Give unadjusted estimates and, if applicable, confounder-adjusted estimates and their precision (eg, 95% confidence interval). Make clear which confounders were adjusted for and why they were included	NA
		(*b*) Report category boundaries when continuous variables were categorized	Table 6-9
		(*c*) If relevant, consider translating estimates of relative risk into absolute risk for a meaningful time period	NA
Other analyses	17	Report other analyses done—eg analyses of subgroups and interactions, and sensitivity analyses	Fig 1
**Discussion**			
Key results	18	Summarise key results with reference to study objectives	Paragraph 1-13
Limitations	19	Discuss limitations of the study, taking into account sources of potential bias or imprecision. Discuss both direction and magnitude of any potential bias	Section 4.1
Interpretation	20	Give a cautious overall interpretation of results considering objectives, limitations, multiplicity of analyses, results from similar studies, and other	Paragraph 10-13, Section 4.1

		relevant evidence	
Generalisability	21	Discuss the generalisability (external validity) of the study results	Paragraph 16, Conclusions
**Other information**			
Funding	22	Give the source of funding and the role of the funders for the present study and, if applicable, for the original study on which the present article is based	NA

*Give information separately for cases and controls in case-control studies and, if applicable, for exposed and unexposed groups in cohort and cross-sectional studies.

Note: An Explanation and Elaboration article discusses each checklist item and gives methodological background and published examples of transparent reporting. The STROBE checklist is best used in conjunction with this article (freely available on the Web sites of PLoS Medicine at http://www.plosmedicine.org/ , Annals of Internal Medicine at http://www.annals.org/, and Epidemiology at http://www.epidem.com/). Information on the STROBE Initiative is available at www.strobe-statement.org.
